# Comparison of Highly Resolved Model-Based Exposure Metrics for Traffic-Related Air Pollutants to Support Environmental Health Studies

**DOI:** 10.3390/ijerph121215007

**Published:** 2015-12-08

**Authors:** Shih Ying Chang, William Vizuete, Michael Breen, Vlad Isakov, Saravanan Arunachalam

**Affiliations:** 1 Institute for the Environment, University of North Carolina at Chapel Hill, 100 Europa Drive, Suite 490, Chapel Hill, NC 27517, USA; changsy5@live.unc.edu; 2 Department of Environmental Sciences and Engineering, University of North Carolina at Chapel Hill, Chapel Hill, NC 27599, USA; airquality@unc.edu; 3 National Exposure Research Laboratory, U.S. Environmental Protection Agency, 109 T.W. Alexander Drive, Research Triangle Park, NC 27711, USA; breen.michael@epa.gov (M.B.); Isakov.vlad@epa.gov (V.I.)

**Keywords:** traffic related air pollution, exposure error, air quality model, space-time kriging, exposure metric, dispersion model

## Abstract

Human exposure to air pollution in many studies is represented by ambient concentrations from space-time kriging of observed values. Space-time kriging techniques based on a limited number of ambient monitors may fail to capture the concentration from local sources. Further, because people spend more time indoors, using ambient concentration to represent exposure may cause error. To quantify the associated exposure error, we computed a series of six different hourly-based exposure metrics at 16,095 Census blocks of three Counties in North Carolina for CO, NO*_x_*, PM_2.5_, and elemental carbon (EC) during 2012. These metrics include ambient background concentration from space-time ordinary kriging (STOK), ambient on-road concentration from the Research LINE source dispersion model (R-LINE), a hybrid concentration combining STOK and R-LINE, and their associated indoor concentrations from an indoor infiltration mass balance model. Using a hybrid-based indoor concentration as the standard, the comparison showed that outdoor STOK metrics yielded large error at both population (67% to 93%) and individual level (average bias between −10% to 95%). For pollutants with significant contribution from on-road emission (EC and NO*_x_*), the on-road based indoor metric performs the best at the population level (error less than 52%). At the individual level, however, the STOK-based indoor concentration performs the best (average bias below 30%). For PM_2.5_, due to the relatively low contribution from on-road emission (7%), STOK-based indoor metric performs the best at both population (error below 40%) and individual level (error below 25%). The results of the study will help future epidemiology studies to select appropriate exposure metric and reduce potential bias in exposure characterization.

## 1. Introduction

Accurate exposure estimation for air pollutants is essential for environmental health studies. In these studies, exposure to air pollutants are often estimated based on ambient concentration level [1,2,3,4]. Ambient concentration collected from fixed-site monitors, for example, provides regional concentration and can be used to determine inter-city difference [5]. Fixed-site monitors, however, are often spatially limited and thus are more suitable for pollutants that are distributed homogenously across space. For pollutants with local sources such as on-road vehicular emission, the data from fixed-site monitors can fail to capture the intra-urban variation [6] resulting in exposure misclassification [7].

A more accurate method for estimating personal exposure is with direct measurement using personal sampling devices [8,9]. For example, Delfino *et al.* [10] compared the association between the reduction in forced expiratory volume in the first second (FEV1) of asthmatic children and four particulate matter (PM) exposure metrics: personal sampling device, indoor concentration at home, outdoor concentration at home, and data from central monitor. The results showed that the reduction in FEV1 is more strongly associated with personal PM exposure or concentration collected indoor at home than concentration collected at a central monitoring site or outdoor concentration at home. Although personal sampling devices can best represent total exposure, they are costly and introduce participant burden for individuals in health studies. For example, a health study conducted in North Carolina, called the Coronary Artery Disease and Environmental Exposure (CADEE), investigated the relationship between personal exposure to multiple air pollutants and adverse health effects. In CADEE, instead of a personal sampling device, the participants wore a personal global positioning system (GPS) device to track their geographical location. To estimate individual exposure with their geographical information, an accurate concentration field is required.

One approach that could be used in the CADEE study is the use of model-based exposure metrics that are less costly and that can cover a wider spatial domain. These exposure metrics can be obtained from various approaches such as space-time kriging, air quality modeling, and land use regression. These approaches, when compared to fixed-site monitors, can increase the potential in predicting intra-urban spatial variability. Space-time kriging technique interpolates observational data to provide spatiotemporally refined concentrations [11,12]. These estimated concentrations can then be used to relate to adverse health effects [13,14,15]. Nevertheless, studies have found that the accuracy of space-time kriging is affected by the location of the available monitors. When estimated locations are far away from the monitors, the resultant concentration estimate is less accurate [16]. Further, space-time kriging may fail to locate the concentration hotspot without adequate monitors for pollutants with local sources, such as on-road vehicular emission that decays to background level within a few hundred meters from roadways [17]. To capture these pollutants, other approaches such as land use regression [18,19] or air quality models [20,21] at a fine spatial resolution are needed.

Although ambient concentration is widely used in health studies as an exposure metric, certain studies have found that indoor concentrations would be a better exposure metric due to time spent indoors [8,9,10]. The Windsor, Ontario Exposure Assessment study has shown that children spend on average more than 67% of their time indoors and receive more than 50% of their PM_2.5_ exposure while indoors [22]. Also, previous studies have pointed out that the variation of air exchange rate (the rate that indoor air is exchanged with outdoor air) can further explain the difference in ozone mortality coefficient across cities [23] and acute air pollution-related morbidity [24] than using outdoor air pollutant concentration from central monitoring sites alone. The approaches for modeling indoor concentration have been developed and evaluated [25,26,27] primarily for subject-specific health study [28]. To our knowledge, these approaches have not been used to provide spatial and temporally refined estimates for predicting personal exposure because house-by-house information required for predicting air exchange rate (AER) is difficult to obtain in a large domain without house-by-house survey.

This paper develops model-based exposure metrics during the CADEE study period so they can be applied for the epidemiologic analysis in the future, taking advantages of the GPS data. Exposure metrics calculated using the “traditional way” were compared with an alternative method. We used public accessible data to gather information required to compute hourly AER at Census block level. These highly resolved AER are than combined with regional background estimates, on-road emissions, and indoor infiltration to create highly resolved indoor concentration field. Our modeling approach complements population-level exposure models (e.g., Stochastic Human Exposure and Dose Simulation (SHEDS) [29], Air Pollutants Exposure Model (APEX) [30,31]), which predict distributions reflecting exposure variability for demographic groups (e.g., school-age children) rather than for specific individuals by using population-level inputs from other studies [29]. We compare these exposure metrics to determine the advantage of providing more details in exposure characterization and quantify the potential exposure error if using a lower tier of exposure metric.

## 2. Experimental Section

### 2.1. Study Design

This analysis focused on three Counties (Durham, Orange, and Wake) in NC that contain two major cities (Durham and Raleigh) and some rural areas (Figure S1), which matched the spatial domain of the CADEE health study. To avoid the exposure misclassification associated with coarse modeling resolution [32,33], hourly concentration were modeled at Census block centroids, resulting in a total of 16,095 concentration receptors. Outdoor and indoor concentrations during the year of 2012 were modeled for PM_2.5_, elemental carbon (EC), CO, and NO*_x_* on an hourly or daily basis. We computed six exposure metrics including: (1) outdoor STOK: outdoor background concentration from space-time ordinary kriging (STOK); (2) indoor STOK: STOK-based indoor concentration; (3) outdoor on-road: outdoor on-road concentration using Research LINE source dispersion model (R-LINE); (4) indoor on-road: on-road-based indoor concentration; (5) outdoor hybrid: outdoor hybrid concentration combing outdoor background and on-road concentration; and (6) indoor hybrid: hybrid-based indoor concentration. The metrics and description is summarized in Table 1. The details for each metric are described in the sections below. We compared the spatial and temporal variability between the six exposure metrics and quantified the potential exposure error at both the population and individual level using the sixth metric as the standard.

**Table 1:** Exposure metrics included in this study.

Metric	Description
Outdoor metrics	
Outdoor STOK	Background concentration obtained from STOK.
Outdoor on-road	Concentration from on-road vehicular emission modeled with R-LINE.
Outdoor hybrid	Summation of outdoor STOK and outdoor on-road
Indoor metrics	
Indoor STOK	Indoor concentration obtained from Equation (1) using outdoor STOK as input
Indoor on-road	Same as above using outdoor on-road as input
Indoor hybrid	Same as above using outdoor hybrid as input

### 2.2. Outdoor Background Concentration

We used space-time ordinary kriging (STOK) to estimate background concentration. STOK uses available monitoring data from U.S. Environmental Protection Agency’s (EPA) Air Quality System (AQS) to interpolate observational data at Census-block centroids. This technique assumes that the concentration value at each estimation point is a linear combination of nearby “hard data” (*i.e.*, the observational data). The linear combination, also known as kriging weight, is determined by minimizing the estimation variance while satisfying the unbiased constraint. The STOK technique is implemented with BMElib (Bayesian Maximization Entropy library) [34]. A detailed description of the STOK algorithm, which was developed and applied for the Near-road Exposures to Urban Air Pollutants Study (NEXUS) [28] in Detroit, Michigan to obtain regional background concentrations can be found in Arunachalam *et al.* [12].

STOK estimates the concentration based on the spatial and temporal covariance between concentrations obtained from different monitoring sites [35]. To obtain a meaningful covariance, the distance between each monitor needs to cover a wide spatial range (from near to far). Due to the limited amount of available monitors in the three-county region in NC, we included monitors in surrounding counties and States for STOK estimation. As a result, for CO, NO*_x_*, PM_2.5_, and EC, there were 48, 33, 103 and 27 available monitors. For EC, since only daily concentration is available, the background concentration is also estimated for a corresponding daily period. For CO, NO*_x_*, and PM_2.5_, the estimation is hourly.

### 2.3. Outdoor on-Road Concentration

We predicted concentration from on-road vehicles using R-LINE [36]. R-LINE is a line source dispersion model that treats roadways as line sources and deploys new formulations for horizontal and vertical plume spread to address the under-prediction in maximum concentration under meteorologically neutral and stable condition [37]. R-LINE requires various inputs including emission, receptor location, and meteorological data.

For developing emission inputs for R-LINE, we adopted a “bottom-up” approach [21] to develop the emission from roadways. The roadway information was collected from Federal Highway Administration’s (FHWA) Freight Analysis Framework version 3 (FAF3) [38], which contains primary and secondary roadways including data on vehicle speed, vehicle type, and annual average daily traffic (AADT) for all vehicles (including passenger and commercial vehicles). Because FAF3 does not provide temporally resolved traffic activity data, temporal allocation factors from EPA’s National Emission Inventory (NEI) were used to allocate AADT to hourly level. This hourly resolved traffic volume was then combined with MOtor Vehicle Emission Simulator (MOVES 2010b) emission factor tables by matching vehicle speed, vehicle type, and road type to calculate emissions. Detailed description about the datasets used to develop emissions inputs for R-LINE can be found in another recent study by the authors [39].

The meteorological data were collected from four nearby National Weather Service (NWS) stations: Raleigh Durham International airport, Rocky Mount-Wilson airport, Chapel Hill Horace Williams airport, and Burlington-Alamance airport. We used AERMINUTE to process 1-min wind speed data from these stations, followed by American Meteorological Society/Environmental Protection Agency Regulatory Model (AERMOD) meteorological processor AERMET (version 14134) to provide necessary meteorological inputs for the dispersion calculations. The receptors were set at Census block centroids within the modeling domain. Each centroid was mapped to the four NWS stations and the site that yielded the shortest distance was chosen to provide meteorological information. Therefore, there are a total of four receptor groups. For each receptor group, all primary and secondary roadways within 50 km were included as emission source.

### 2.4. Outdoor Hybrid Concentration

We combined the outdoor background concentration (from STOK) and outdoor on-road concentration (from R-LINE) to calculate a spatially and temporally refined concentration field in the three-County region. The background concentration in this study was defined as the regional concentration that would be measured if local sources were zeroed out. Therefore, it is not influenced by local sources but represents a large-scale overall pattern. A similar approach was used in U.S. EPA’s National Air Toxics Assessments (NATA) [40] where observations from AQS sites were used to provide background, and wherein, the quality of the collected ambient monitoring data was used to determine background concentration in three slightly different ways. The method to obtain hybrid concentrations is similar to another study by the same authors [39]. The local source we considered in this study was on-road mobile sources, which have great variation in emissions and is influenced by the meteorology at a local scale. The sum of outdoor background concentration and outdoor on-road concentration were computed hourly at Census block centroids. Note that, because EC only has daily background concentration, the hourly resolution feature is from on-road concentration alone.

### 2.5 Indoor Concentration and Air Exchange Rate

We used a mass balance differential equation [27] to describe the change in indoor concentration:

(1) $$\frac{dC_{in}}{dt}=P\times AER\times C_{out}-\left(AER+k_{d}\right)\times C_{in}$$

where *C_out_* and *C_in_* are the outdoor concentration and indoor concentration in μg/m^3^, t is time in hour (h), *P* is the dimensionless penetration factor, *k_d_* is the deposition rate in h^−1^. The first term of the equation (*P* × *AER* × *C_out_*) represents the penetration process from outdoor to indoor and the second term ((AER + *k_d_*) × *C_in_*) represents the removal of indoor concentration by AER and indoor deposition. The penetration factor and deposition rate for each pollutant were set to reported literature values shown in Table 2. For the three outdoor concentrations (background, on-road, and hybrid), we used Equation (1) to calculate their corresponding indoor concentration. Because on-road concentration varies substantially across time, we used the dynamic mass balance model (Equation (1)) rather than assuming steady state conditions [41].

We used MATLAB’s (version R2013a, MathWorks Inc., Natick, MA, USA) differential equation solver, *ode15s*, to solve Equation (1) to obtain indoor concentration. The solver was set to report the indoor concentration for each hour. For each hour, the indoor concentration from the previous hour was used as the initial value. The initial indoor concentration for the first hour was assumed to be zero. This causes only a modest impact on the analysis because the model is stabilized within the first two to three hours. There were three types of outdoor concentration for Equation (1): STOK, on-road, and hybrid. For each type of outdoor concentration, Equation (1) was used to obtain the corresponding indoor concentration.

**Table 2:** Penetration factor *P* and deposition rate *k_d_*.

Pollutant	Penetration Factor	Deposition Rate (h^−1^)	Source
CO	1	0	Dionisio *et al.* [42]
NO*_x_*	1	0.5	Weschler *et al.* [43]
PM_2.5_	0.84	0.21	Breen *et al.* [44]
EC	0.98	0.29	Meng *et al.* [45]

We calculated hourly AER for 10 randomly sampled houses within each Census block, and then averaged them to represent that Census block. The AER was computed using the mechanistic Lawrence Berkeley Laboratory (LBL) AER model [46]. The LBL model assumes the building to be a single and well-mixed compartment [47]. The LBL model calculates the airflow rate as:

(2) $$Q_{inf}=A_{inf}\sqrt{k_{s}\left|T_{in}-T_{out}\right|+k_{w}U^{2}}$$

where *Q_inf_* is the airflow rate in L/h, *A_inf_* is the effective air leakage area (in *cm^2^*), *k_s_* is the stack coefficient in $\frac{{\left(\frac{L}{s}\right)}^{2}}{\left(cm^{4}\cdot K\right)}$, *k_w_* is the wind coefficient $\frac{{\left(\frac{L}{s}\right)}^{2}}{\left(cm^{4}\cdot {\left(\frac{m}{s}\right)}^{2}\right)}$, *T_in_* and *T_out_* are the indoor and outdoor temperatures in °C, and *U* is the wind speed in m/s. The AER is calculated as:

(3) $$AER=\frac{Q_{inf}}{V}$$

where *V* is the house volume in L.

We followed Breen *et al.* [46] to determine the input parameters of Equation (2). Breen *et al.* [46] compared AER predictions to data from 642 daily AER measurements across 31 detached homes during each of four seasons in central North Carolina. For individual model-predicted and measured AER, the median absolute difference was 43% (0.17 h^−1^) [44]. *k_s_* and *k_w_* were set to reported literature values based on house-specific information including house height and local sheltering (Tables S1 and S2). *T_out_* and *U* were obtained from the NWS sites as described in the outdoor on-road concentration section. *T_in_* was set at 23.6 °C, which is the average indoor temperature measured in this region from Breen *et al.* (2010) [46].

To determine *A_inf_*, we used a leakage area model, which was previously evaluated in another study [48] and was found to perform well with fewer input parameters, because information on air leakage through floors is not available. *A_inf_* is calculated as:

(4) $$A_{inf}=\frac{NL}{NF}$$

where *NL* is the normalized leakage and *NF* is the normalization factor (cm^−2^). The *NL* is dimensionless and was calculated based on a regression model with construction year and floor area as predicting variable. The *NL* is calculated as:

(5) $$NL=\text{exp}\left(\beta_{0}+\beta_{1}Y_{built}+\beta_{2}A_{floor}\right)$$

where *Y_built_* is construction year and *A_floor_* is the floor area in m^2^. β_0_, β_1_ and β_2_ are the regression parameters, which were set at literature reported values for low-income homes (β_0_ = 11.1, β_1_ = −5.37 × 10^−3^, and β_2_ = −4.18 × 10^−3^ m^−2^) and conventional homes (β_0_ = 20.7, β_1_ = −1.07 × 10^−2^, and β_2_ = −2.20 × 10^−3^ m^−2^). As previously reported, the *NL* model was fit to a national database of leakage areas for 70,000 homes across 30 states in the Midwest (most-sampled region), West, South, and Northeast (least-sampled region), which included residences with household incomes below 125% of the poverty guideline [48]. The parameters were estimated by Chan *et al.* [48] from homes built between 1895 and 2000, which is similar to the homes in this study that were built between 1700 and 2015. The *NF* is calculated as:

(6) $$NF=\frac{1000}{A_{floor}}{\left(\frac{H}{2.5}\right)}^{0.3}$$

where *H* is the building height in meters.

Equations (2)–(6) require inputs including *H*, *A_floor_*, and *Y_built_*. Further, the required parameters (*k_s_*, *k_w_*, β_0_, β_1_, and β_2_) need to be determined by additional information including household income, shelter class, and number of stories. To obtain *A_floor_* and *Y_built_*, we relied on the three Counties’ real estate property data. Because the real estate property data also include apartments, for which the LBL doesn’t apply, we remove buildings with floor area greater than 7000 square feet (possible multiunit apartments), resulting in approximately 370,000 houses in the modeling domain. *H* was calculated based on number of stories, where each story was assumed to be 2.5 m and adding an additional 0.5 m for roof space. The number of stories is reported in the real estate property data of Wake County but not for Durham and Orange Counties. For these two Counties, we followed Chan *et al.* [48] to set houses with floor area less than 1000 m^2^ at one story and those greater at two stories. This uncertainty does not constitute a large source of error in estimating NL, because NL only varies in proportion to *H*^0.3^ [48]. The household income distribution was obtained from the U.S. Census Bureau’s American Community Survey (ACS) 2013 [49]. Because this dataset only contains household income distribution at Census block group level, we calculated the fraction of houses below 125% of poverty line within each Census block group then randomly sample from this fraction to determine the household income status for a sampled house. The shelter class for each sampled house was determined based on the house density of each Census block. The house density for each Census block was calculated and the cutoff values for each shelter class were determined from aerial and street-level images in Google map’s satellite view. The cutoff density is summarized in Table S3.

### 2.6. Data Analysis

For each exposure metric, we computed the normalized difference and normalized absolute difference to represent individual exposure difference using hybrid-based indoor concentration as standard. The normalized difference was defined as:

(7) $$ND=\frac{C_{x}-C_{s}}{C_{s}}\times 100\%$$

where *ND* is normalized difference, *C_X_* is the lower tiered exposure metrics, and *C_s_* is the standard exposure metric (hybrid-based indoor concentration). Normalized absolute difference (NAD) was defined as:

(8) $$NAD=\frac{\left|C_{x}-C_{s}\right|}{C_{s}}\times 100\%$$

We calculated both *ND* and *NAD* since *ND* indicates the direction of bias (*i.e.*, overestimation or underestimation), whereas *NAD* indicates the magnitude of deviation. To compare the temporal and spatial variability of different exposure metrics across pollutants, we computed the coefficient of variation (CV), which was defined as:

(9) $$CV=\frac{\sigma }{\mu }$$

where σ is the standard deviation of concentration and μ is the mean concentration [7]. *CV* is a dimensionless indicator that normalized the variation from the effect of concentration magnitude for different pollutant. The higher the *CV*, the higher the degree of variability is in concentration. The temporal *CV* was defined as the *CV* calculated across hours, with one temporal *CV* for each Census block (*n* = 16,095) for each pollutant and each metric. The spatial *CV* was defined as the *CV* calculated across Census blocks, with one spatial *CV* for each hour (*n* = 8784) for each pollutant and each metric.

## 3. Results

To assess the impact from the additional parameters (on-road component and indoor infiltration) on STOK, we present our data considering one parameter at a time in each of the first three sub-sections below. In Section 3.2 and Section 3.3, we summarize the potential exposure error at population and individual level. Given the multiple models and pollutants discussed below, we have underscored the phrases: outdoor STOK, outdoor on-road, outdoor hybrid, indoor STOK, indoor on-road and indoor hybrid, and italicized the statistical indicators (*spatial CV*, *temporal CV*, *ND*, and *NAD*) and the pollutant names (*CO*, *NO_x_*, *PM_2.5_* and *EC*) throughout this section, for ease of readability.

### 3.1. The Effect of on-Road Component

**Figure 1:**
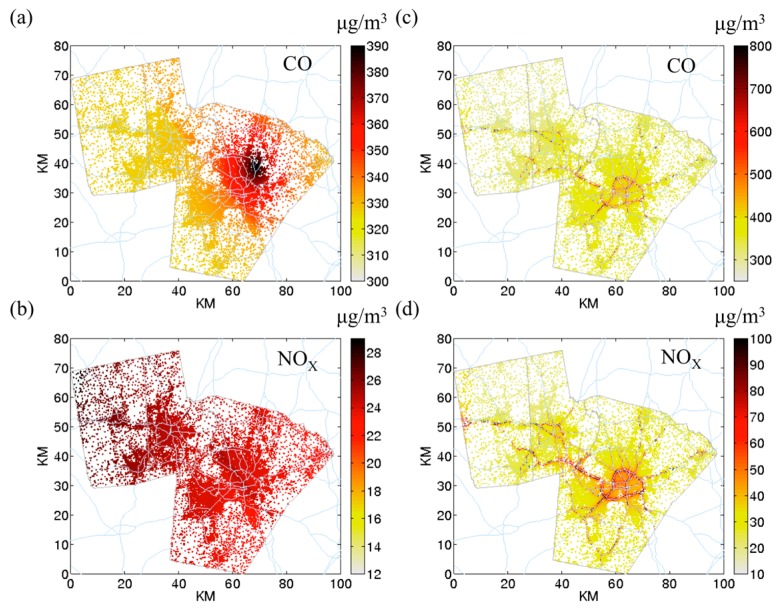
Outdoor concentration of *CO* and *NO_x_* at Census block centroids at 07:00. (**a**) *CO* outdoor space-time ordinary kriging (STOK); (**b**) *NO_x_* outdoor STOK; (**c**) *CO* outdoor hybrid; (**d**) *NOx* outdoor hybrid. The color bar represents concentration in μg/m^3^. (Note that the color scale is different for the four figures to emphasize the concentration ranges that vary by pollutant).

Figure 1 shows the outdoor STOK and outdoor hybrid concentration maps for *CO* (Figure 1a,c) and *NO_x_* (Figure 1b,d) at Census block centroids for four different metrics. We presented morning traffic peak hour (07:00) because the on-road contribution is the greatest. At 07:00, concentration from roadways is clearly seen with outdoor hybrid (Figure 1c,d) but not outdoor STOK (Figure 1a,b). Note the color scale is different among the four figures to properly display the data. STOK cannot capture the near road concentrations because there is a limited amount of available monitors in this region. Further, the location of monitors is crucial for STOK to estimate the concentration. *CO* has a “kriging island” (*i.e.*, a concentration hotspot surrounding a monitor, Figure 1a) but not for *NO_x_* (Figure 1b).

**Figure 2:**
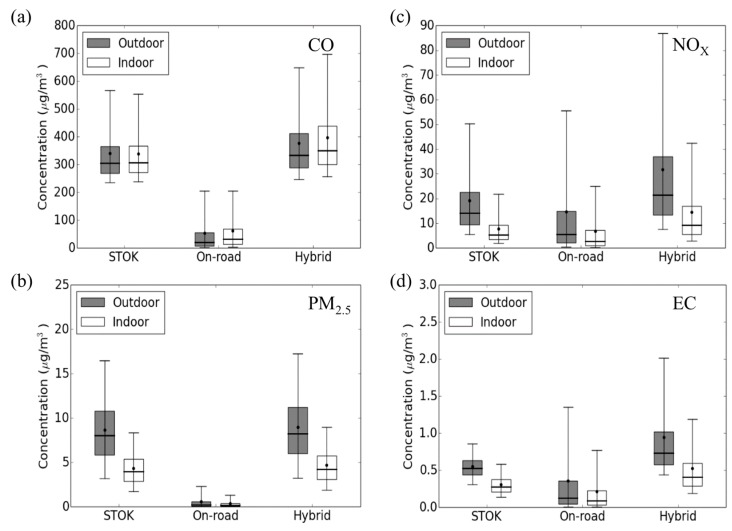
Hourly pollutant concentration for each Census block in Durham, Orange, and Wake Counties, North Carolina (NC) in 2012. (**a**) *CO*; (**b**) *PM_2.5_*; (**c**) *NO_x_*; and (**d**) *EC*. Bottom and top of box represents 25th and 75th percentiles, the line in the middle of the box is the median, the ends of the whisker are the 5th and 95th percentiles, and the dot on the whisker is the mean.

Figure 2 shows the hourly concentration boxplot for the four pollutants under different exposure metrics. For outdoor *CO* and *PM_2.5_*, the major contributor to the outdoor hybrid is the outdoor STOK. For *CO* (Figure 2a), the average outdoor STOK (340.45 μg/m^3^) is 6.23 times higher than the average outdoor on-road (54.67 μg/m^3^). For *PM*_2.5_ (Figure 2b), the average outdoor STOK (8.69 μg/m^3^) is 14.02 times higher than the average outdoor on-road (0.62 μg/m^3^). For these two pollutants, because the outdoor STOK dominates the hybrid concentration, the outdoor hybrid is less different from the outdoor STOK concentration.

For *NO_x_* and *EC*, both outdoor STOK and outdoor on-road contribute significantly to the outdoor hybrid. For *NO_x_* (Figure 2c), although the average outdoor STOK (19.24 μg/m^3^) is 32% higher than the outdoor on-road (14.63 μg/m^3^), the upper 95% bound of outdoor on-road (55.6 μg/m^3^) is 10% higher than the outdoor STOK (50.33 μg/m^3^). For *EC* (Figure 2d), the average outdoor STOK (0.55 μg/m^3^) is 52% higher than the outdoor on-road (0.36 μg/m^3^) but the upper 95% bound of outdoor on-road (1.35 μg/m^3^) is 57% higher than the outdoor STOK (0.86 μg/m^3^). As a result, for these two pollutants, the average outdoor hybrid is 65% and 72% higher than the average outdoor STOK for *NO_x_* and *EC*.

**Figure 3:**
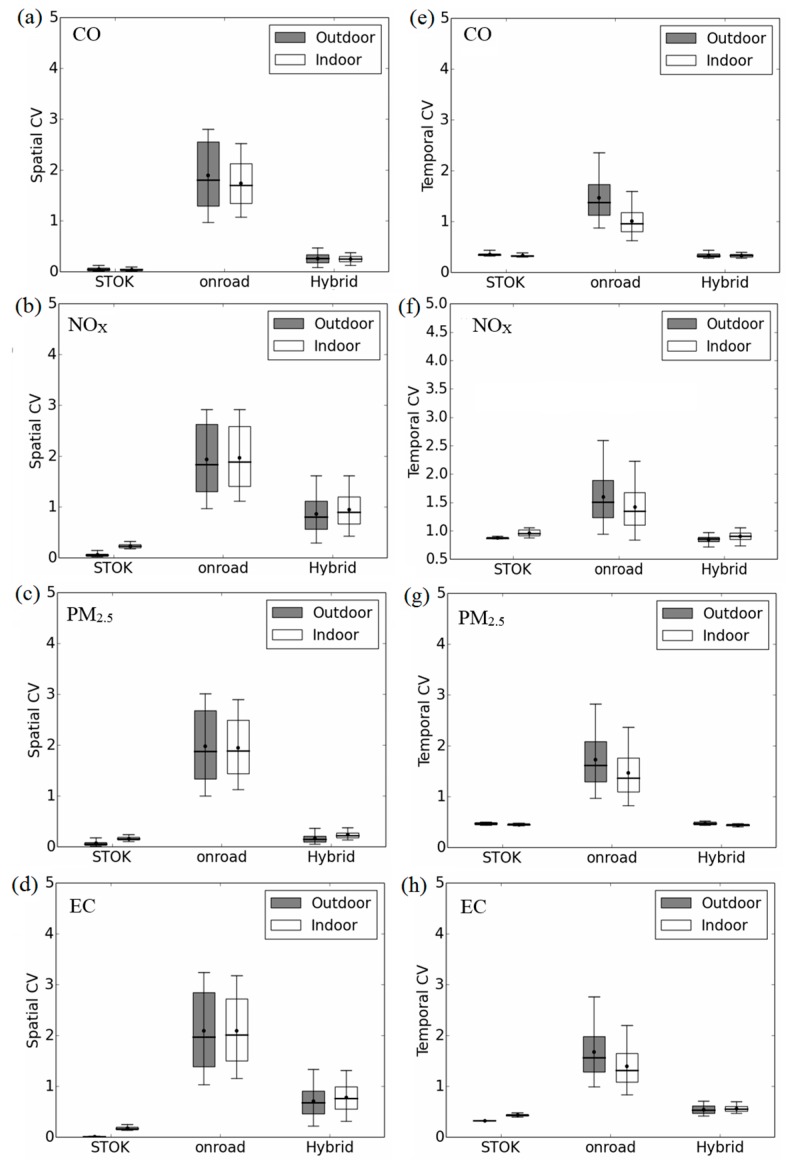
*Spatial CV* for each hour (**left panel**); and *Temporal CV* for each Census block (**right panel**) for *CO* (**a**,**e**); *NOx* (**b**,**f**); *PM_2.5_* (**c**,**g**); and *EC* (**d**,**h**). Bottom and top of box represents 25th and 75th percentiles, the line in the middle of the box is the median, the ends of the whisker are the 5th and 95th percentiles, and the dot on the whisker is the mean.

As shown in Figure 1 and the wider range for the outdoor hybrid compared to outdoor STOK in Figure 2 (dark boxes), adding the outdoor on-road introduces different spatial variability for different pollutants. Figure 3 left panel quantifies the spatial component of this variability using *spatial CV*. For all pollutants, the outdoor on-road shows a great spatial variability (average *spatial CV* ~2). As a result, for the pollutants that have large contribution from outdoor on-road concentration (38% for *NO_x_* and 46% for *EC*), the outdoor hybrid would yield much higher spatial variation (average *spatial CV* = 0.87 for *NO_x_* and 0.71 for *EC*) than outdoor STOK (average *spatial CV* = 0.065 for *NO_x_* (Figure 3b) and 0.014 for *EC* (Figure 3d)). It is worth noticing that although *CO* and *PM_2.5_* in this region is dominated by background concentration, adding outdoor on-road can still increase the spatial variability (average *spatial CV* from 0.06 for outdoor STOK to 0.26 for outdoor hybrid for *CO* and 0.07 for outdoor STOK to 0.17 for outdoor hybrid for *PM_2.5_*), indicating the importance of the on-road emission for the near-road environment even when the contribution is relatively small (14% for *CO* and 7% for *PM_2.5_*). Corroborating illustrations are shown in the authors’ peer-reviewed paper [39] where the hybrid contribution for *PM_2.5_* drops by 20% within 150 meters from roadways.

*Temporal CV* is summarized in Figure 3 right panel. The outdoor on-road shows a great temporal variation (average *temporal CV* ~1.5, (Figure 3, dark boxes for outdoor on-road)). This high temporal variation is from the bottom up approach used in the R-LINE modeling where the temporal pattern of on-road emission is captured. For *CO* and *PM_2.5_* (Figure 3e,g), the outdoor hybrid yields similar average *temporal CV* to outdoor STOK because for these two pollutants, outdoor STOK dominates the total concentration. Therefore, although outdoor on-road shows large temporal variation, the variation is lost after outdoor on-road and outdoor STOK are combined for *CO* and *PM_2.5_*. For *NO_x_* (Figure 3f), although 38% of the outdoor hybrid is from the outdoor on-road, because the outdoor on-road only affects Census blocks within a few hundred meters from roadways, the overall *temporal CV* for outdoor hybrid is less different from the outdoor STOK. For *EC*, because the outdoor on-road contributes 46% to the outdoor hybrid, the average *temporal CV* increases by 72% from 0.33 for outdoor STOK to 0.57 for outdoor hybrid (Figure 3h).

**Figure 4:**
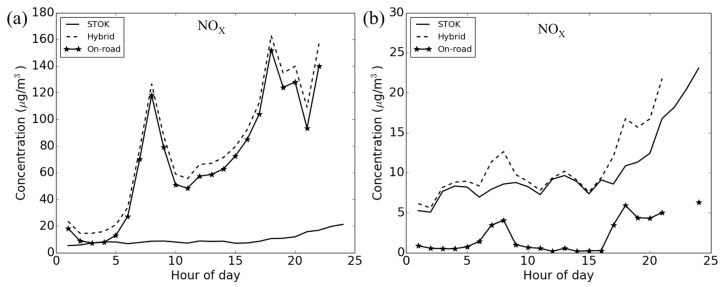
Time series plot on January 3rd for *NO_x_* at (**a**) a near-road Census block (14.1 m from roadway); and (**b**) a remote Census block (9.6 km from roadway).

For the *temporal CV*, only the Census blocks near roadways would be affected by outdoor on-road. Examples for *NO_x_* are shown in Figure 4 with a Census block that is 14.1 m from a roadway (left panel) and a Census block that is 9.6 km from a roadway (right panel) and comparing concentrations at each of the two locations for a day At the near-road Census block (Figure 4a), outdoor on-road contributes, on average, 89% to outdoor hybrid. The contribution from outdoor on-road is the greatest (over 90%) during morning (07:00 to 09:00) and afternoon (17:00 to 19:00) traffic peak hours and the *temporal CV* increases by 40% (from 0.42 for outdoor STOK to 0.59 for outdoor hybrid). On the other hand, at a remote Census block (Figure 4b), the outdoor on-road for *NO_x_* contributes, on average, only 18% to the outdoor hybrid. As a result, the *temporal CV* only increases slightly by 7% from 0.44 for outdoor STOK to 0.47 for outdoor hybrid. All the other pollutants show a similar pattern as *NO_x_* (Figure S2).

The effect of on-road component on indoor metrics shows similar pattern to that of outdoor metrics (White-colored boxes from Figure 3e,h). We present the difference between outdoor and indoor metrics in the next section.

### 3.2. The Effect of Indoor Infiltration

**Figure 5:**
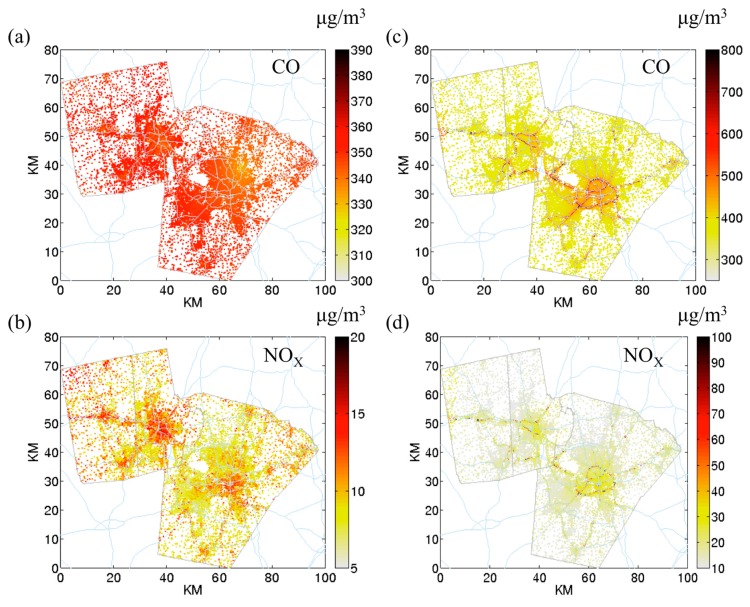
Indoor concentration of *CO* and *NO_x_* at 07:00 with (**a**) *CO* indoor STOK; (**b**) *NO_x_* indoor STOK; (**c**) *CO* indoor hybrid; (**d**) *NO_x_* indoor hybrid. The color bar represents concentration in μg/m^3^ (Note that the color scale is different for the four figures to emphasize the concentration ranges that vary by pollutant).

**Figure 6:**
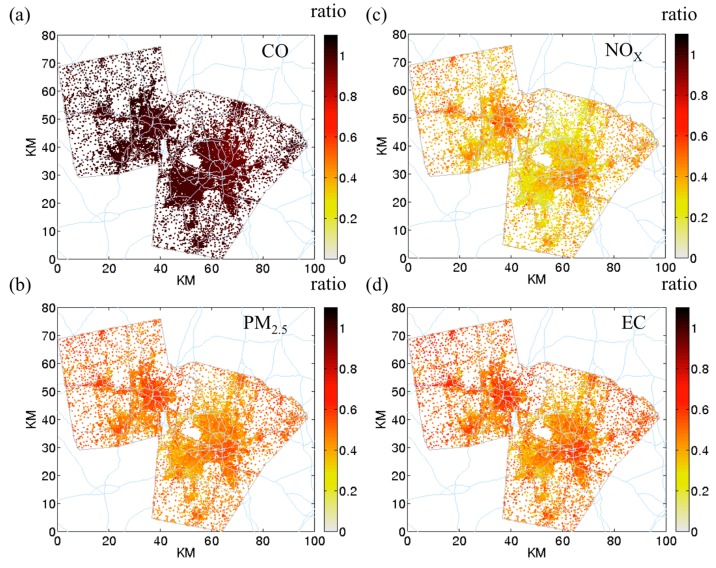
Indoor-outdoor concentration ratio using mean hybrid concentration at 07:00. (**a**) *CO*; (**b**) *PM_2.5_*; (**c**) *NO_x_*; and (**d**) *EC*.

Figure 5 shows the indoor concentration for *CO* and *NO_x_*. At 07:00, the spatial pattern for indoor metrics is similar to the outdoor metrics (Figure 1) except for indoor STOK *NO_x_* (Figure 5c). The extra spatial variation for indoor STOK *NO_x_* shows a similar spatial pattern to AER (Figure S3). However, this pattern is not seen for *CO* (Figure 5a). On average, compared to the outdoor concentration, the indoor concentration is 66% lower for *NO_x_*, 46% lower for *PM_2.5_*, and 43% lower for *EC* (Figure 2b–d). *CO* on the other hand, shows a slightly higher (5.9%) indoor concentration than outdoor concentration (Figure 2a). This is because of the relatively high penetration factor (1) and low indoor deposition rate (0 h^−1^) for *CO*, resulting in the accumulation for indoor concentration. However, in general, *CO* is not affected by the indoor infiltration. Figure 6 shows the concentration ratio at 07:00 between indoor and outdoor hybrid concentration. 

For *PM_2.5_* and *EC* (Figure 6b,d), the ratio is ~0.7 and for *NO_x_* (Figure 6c), the ratio is ~0.5. The difference is because of the higher indoor deposition for *NO_x_* (0.5 h^−1^) compared to *PM_2.5_* (0.21 h^−1^) and *EC* (0.29 h^−1^). The high ratio area overlaps with the area with high AER. High AER is seen mostly in urban area. As these areas usually have higher density of roadways, the residents have the potential to be exposed to higher air pollutant concentrations in the indoor environment.

The *spatial CV* for indoor STOK is higher than outdoor STOK (Figure 3 left panel). Because outdoor STOK is homogenously distributed across space, pollutants with higher indoor deposition rate (*i.e.*, *NO_x_*, *PM_2.5_*, and *EC*) have a higher average *spatial CV* in indoor STOK than in outdoor STOK. Compared to the outdoor STOK, the average *spatial CV* of the indoor STOK is 3.6 fold higher for *NO_x_*, 2.2 fold higher for *PM_2.5_*, and 12.9 fold higher for *EC*. As shown in Figure 5b with the example for *NOx*, this increase in spatial variability is from the spatial variation of AER. Indoor on-road’s *spatial CV* is not much different from outdoor on-road. For *NO_x_*, *PM_2.5_*, and *EC*, compared with the mean *spatial CV* of the outdoor on-road, the average *spatial CV* of indoor on-road changes less than 2%. Because the spatial variation for outdoor on-road is large (*spatial CV* ~2), the extra spatial variation from AER is “covered” and the indoor on-road demonstrated similar *spatial CV* to outdoor on-road. For the outdoor hybrid, the effect of infiltration on *spatial CV* depends on the spatial variability of outdoor hybrid. For *NO_x_* and *EC*, because the major contributor for outdoor hybrid is outdoor on-road, the *spatial CV* of outdoor hybrid is high (~0.8). Therefore, the indoor hybrid shows only a slightly higher (10%) *spatial CV* than the outdoor hybrid for *NO_x_* and *EC*. For *PM_2.5_*, because outdoor STOK dominates the outdoor hybrid, the *spatial CV* of outdoor hybrid is low (~0.17) the indoor infiltration produces the indoor hybrid that has higher *spatial CV* (40%) than the outdoor hybrid.

*Temporal CV* in general, does not change much for STOK and hybrid between outdoor and indoor metrics (Figure 3 right panel). For the on-road, due to the accumulation effect mentioned previously, the temporal variation is smoothed out, resulting in a lower temporal variation in indoor metrics than outdoor metrics.

### 3.3. The Overall Effect on Exposure Error

Because people spend more time indoors and STOK cannot capture the impact from a local source, we used the indoor hybrid as a standard to compare to other metrics. To quantify the potential population exposure error using the other metrics, we created contingency tables [33] for each pollutant that compares quintiles of the population exposure for the annual average concentration (Table 3, Table 4, Table 5 and Table 6). These tables’ diagonal values represent the percentage of Census blocks of a metric that agrees with the indoor hybrid. With a perfect agreement with indoor hybrid, the diagonal values would be 100% and the non-diagonal values would be 0. For example, Table 3 shows the contingency table for *CO*. Assuming the indoor hybrid is closer to the actual exposure, the top left entry represents that of the population in the lowest quintile (~3200 Census blocks exposed to 347.4 to 362.1 μg/m^3^ of *CO*), the outdoor hybrid metric correctly classified 91%. For *CO* with the outdoor hybrid, 9% of the Census blocks were grouped to the second lowest group. The high diagonal values for *CO* for the outdoor hybrid metric (>81%) indicate a good agreement between it and the indoor hybrid. It is worth noting that the outdoor STOK metric does not agree well with the indoor hybrid (8% to 34% agreement).

**Table 3:** Contingency table for *CO* showing agreement between exposure quintiles. The values represent percentage of Census blocks in each quintile. Concentration ranges are shown in parentheses. Boxed percentages along diagonals would be 100% for a perfect match.

	Percentile	Concentration (μg/m^3^)	Indoor Hybrid				
			0–20	20–40	40–60	60–80	80–100
			(347.4, 362.1)	(362.1, 372.0)	(372.0, 385.4)	(385.4, 411.6)	(411.6, 2242.2)
Outdoor hybrid	0%–20%	(330.2, 345.9)	91	10	0	0	0
	20%–40%	(345.9, 353.9)	9	81	11	0	0
	40%–60%	(353.9, 365.7)	0	9	85	6	0
	60%–80%	(365.7, 388.0)	0	0	4	92	3
	80%–100%	(388.0, 2024.4)	0	0	0	2	97
Indoor on-road	0%–20%	(6.7, 25.2)	79	21	0	0	0
	20%–40%	(25.2, 35.4)	15	64	21	0	0
	40%–60%	(35.4, 49.2)	6	13	70	11	0
	60%–80%	(49.2, 76.6)	0	2	9	83	6
	80%–100%	(76.6, 1903.4)	0	0	0	6	94
Outdoor on-road	0%–20%	(5.8, 21.3)	78	23	0	0	0
	20%–40%	(21.3, 30.0)	16	61	24	0	0
	40%–60%	(30.0, 42.2)	6	14	66	13	0
	60%–80%	(42.2, 66.5)	0	2	10	82	6
	80%–100%	(66.5, 1694.4)	0	0	0	5	94
Indoor STOK	0%–20%	(317.2, 336.1)	25	16	12	22	25
	20%–40%	(336.1, 339.3)	10	18	18	23	30
	40%–60%	(339.3, 341.3)	12	27	30	17	14
	60%–80%	(341.3, 343.7)	6	16	26	29	23
	80%–100%	(343.7, 347.7)	47	22	14	9	8
Outdoor STOK	0%–20%	(322.3, 337.6)	24	16	12	22	27
	20%–40%	(337.6, 340.7)	12	18	18	24	28
	40%–60%	(340.7, 342.2)	8	27	34	17	15
	60%–80%	(342.2, 343.7)	8	17	23	29	22
	80%–100%	(343.7, 347.7)	48	23	13	8	8

For *NO_x_* and *EC* (Table 4 and Table 6), the outdoor hybrid does not perform well (the agreement is between 33% and 49% for the lower four groups) except for the highest quintile (73% for *NO_x_* and 76% for *EC*). All the other outdoor metrics for *NO_x_* and *EC* perform poorly (Table 4, Table 5 and Table 6). The best agreement for *NO_x_* and *EC* is with the indoor on-road (agreement between 45% and 90%). At the lowest quintile, indoor STOK performs well (68% for *NO_x_* and 69 for *EC*).

For *PM_2.5_* (Table 5), indoor STOK performs the best (agreement between 59% and 90%). All other metrics perform poorly. For all pollutants in general, all outdoor metrics perform relatively poorer than indoor metrics. Outdoor STOK, in specific, performs very poorly (agreement ranges from 8% to 34% considering all pollutants). Since space-time kriging is often used in environmental health studies to quantify air pollutant exposures [13,14,15], this part of analysis shows that there is a great potential for this metric to misclassify exposures for all four pollutants studied.

**Table 4:** Contingency table for *NO_x_* showing agreement between exposure quintiles. The values represent percentage of Census blocks in each quintile. Concentration ranges are shown in parentheses. Boxed percentages along diagonals would be 100% for a perfect match.

	Percentile	Concentration (μg/m^3^)	Indoor Hybrid				
			0–20	20–40	40–60	60–80	80–100
			(3.4, 9.3)	(9.3, 11.1)	(11.1, 13.5)	(13.5, 17.7)	(17.7, 307.9)
Outdoor hybrid	0%–20%	(18.4, 23.3)	49	31	17	3	0
	20%–40%	(23.3, 25.4)	32	34	25	10	0
	40%–60%	(25.4, 28.6)	13	26	33	26	1
	60%–80%	(28.6, 35.1)	4	8	20	41	26
	80%–100%	(35.1, 594.7)	1	1	4	20	73
Indoor on-road	0%–20%	(0.5, 2.6)	69	26	5	0	0
	20%–40%	(2.6, 3.7)	28	49	22	2	0
	40%–60%	(3.7, 5.3)	3	24	55	18	0
	60%–80%	(5.3, 8.9)	0	1	18	70	10
	80%–100%	(8.9, 299.0)	0	0	0	10	90
Outdoor on-road	0%–20%	(1.7, 6.1)	49	32	17	3	0
	20%–40%	(6.1, 8.3)	33	33	26	9	0
	40%–60%	(8.3, 11.4)	13	26	33	26	1
	60%–80%	(11.4, 18.0)	4	8	20	42	25
	80%–100%	(18.0, 577.4)	1	1	4	20	73
Indoor STOK	0%–20%	(2.4, 6.5)	68	18	6	4	4
	20%–40%	(6.5, 7.3)	22	40	20	11	8
	40%–60%	(7.3, 8.2)	9	26	31	21	13
	60%–80%	(8.2, 9.3)	2	13	30	31	24
	80%–100%	(9.3, 14.4)	0	2	13	33	52
Outdoor STOK	0%–20%	(18.9, 19.0)	22	23	24	18	14
	20%–40%	(19.0, 19.3)	13	16	19	22	30
	40%–60%	(19.3, 19.3)	16	15	16	20	31
	60%–80%	(19.3, 19.4)	18	21	21	26	14
	80%–100%	(19.4, 20.2)	31	25	21	14	10

**Table 5:** Contingency table for *PM_2.5_* showing agreement between exposure quintiles. The values represent percentage of Census blocks in each quintile. Concentration ranges are shown in parentheses. Boxed percentages along diagonals would be 100% for a perfect match.

	Percentile	Concentration (μg/m^3^)	Indoor Hybrid				
			0–20	20–40	40–60	60–80	80–100
			(1.72, 4.03)	(4.03, 4.45)	(4.45, 4.83)	(4.83, 5.28)	(5.28, 21.21)
Outdoor hybrid	0%–20%	(7.91, 8.43)	27	24	22	19	8
	20%–40%	(8.43, 8.79)	27	21	20	19	13
	40%–60%	(8.79, 8.90)	26	27	23	15	10
	60%–80%	(8.90, 9.17)	15	19	22	24	20
	80%–100%	(9.17, 34.29)	6	8	13	23	50
Indoor on-road	0%–20%	(0.03, 0.15)	42	26	20	10	2
	20%–40%	(0.15, 0.21)	34	30	19	13	5
	40%–60%	(0.21, 0.29)	16	27	28	22	8
	60%–80%	(0.29, 0.47)	7	13	24	31	26
	80%–100%	(0.47, 16.33)	2	4	11	23	60
Outdoor on-road	0%–20%	(0.07, 0.26)	28	26	24	17	6
	20%–40%	(0.26, 0.34)	33	27	20	15	6
	40%–60%	(0.34, 0.47)	22	25	23	20	9
	60%–80%	(0.47, 0.74)	12	14	20	25	29
	80%–100%	(0.74, 26.00)	5	8	13	23	51
Indoor STOK	0%–20%	(1.67, 3.85)	90	8	1	1	1
	20%–40%	(3.85, 4.21)	11	73	11	3	2
	40%–60%	(4.21, 4.53)	0	19	62	14	5
	60%–80%	(4.53, 4.89)	0	0	25	59	16
	80%–100%	(4.89, 6.55)	0	0	0	24	76
Outdoor STOK	0%–20%	(8.46, 8.61)	15	16	18	23	27
	20%–40%	(8.61, 8.69)	32	27	22	12	7
	40%–60%	(8.69, 8.72)	15	15	17	22	31
	60%–80%	(8.72, 8.76)	18	19	20	23	20
	80%–100%	(8.76, 9.14)	20	23	23	20	14

**Table 6:** Contingency table for *EC* showing agreement between exposure quintiles. The values represent percentage of Census blocks in each quintile. Concentration ranges are shown in parentheses. Boxed percentages along diagonals would be 100% for a perfect match.

	Percentile	Concentration (μg/m^3^)	Indoor Hybrid				
			0–20	20–40	40–60	60–80	80–100
			(0.15, 0.37)	(0.37, 0.43)	(0.43, 0.50)	(0.50, 0.62)	(0.62, 12.74)
Outdoor hybrid	0%–20%	(0.65, 0.74)	49	32	17	2	0
	20%–40%	(0.74, 0.79)	32	35	27	7	0
	40%–60%	(0.79, 0.86)	14	25	34	26	1
	60%–80%	(0.86, 1.01)	4	7	19	46	23
	80%–100%	(1.01, 19.61)	1	1	3	18	76
Indoor on-road	0%–20%	(0.02, 0.09)	65	28	7	0	0
	20%–40%	(0.09, 0.12)	30	45	23	2	0
	40%–60%	(0.12, 0.17)	5	25	52	18	0
	60%–80%	(0.17, 0.28)	0	2	18	69	11
	80%–100%	(0.28, 12.38)	0	0	0	11	89
Outdoor on-road	0%–20%	(0.04, 0.15)	48	32	18	2	0
	20%–40%	(0.15, 0.20)	33	34	26	8	0
	40%–60%	(0.20, 0.28)	14	25	33	26	1
	60%–80%	(0.28, 0.43)	5	7	19	45	23
	80%–100%	(0.43, 19.00)	1	1	3	18	76
Indoor STOK	0%–20%	(0.11, 0.27)	69	17	6	4	4
	20%–40%	(0.27, 0.30)	22	41	18	11	9
	40%–60%	(0.30, 0.33)	8	26	30	20	15
	60%–80%	(0.33, 0.36)	1	13	31	29	25
	80%–100%	(0.36, 0.49)	0	2	15	36	47
Outdoor STOK	0%–20%	(0.55, 0.55)	13	20	22	22	23
	20%–40%	(0.55, 0.55)	10	15	17	22	36
	40%–60%	(0.55, 0.55)	31	26	24	13	6
	60%–80%	(0.55, 0.56)	14	16	20	26	23
	80%–100%	(0.56, 0.56)	31	23	18	16	12

**Figure 7:**
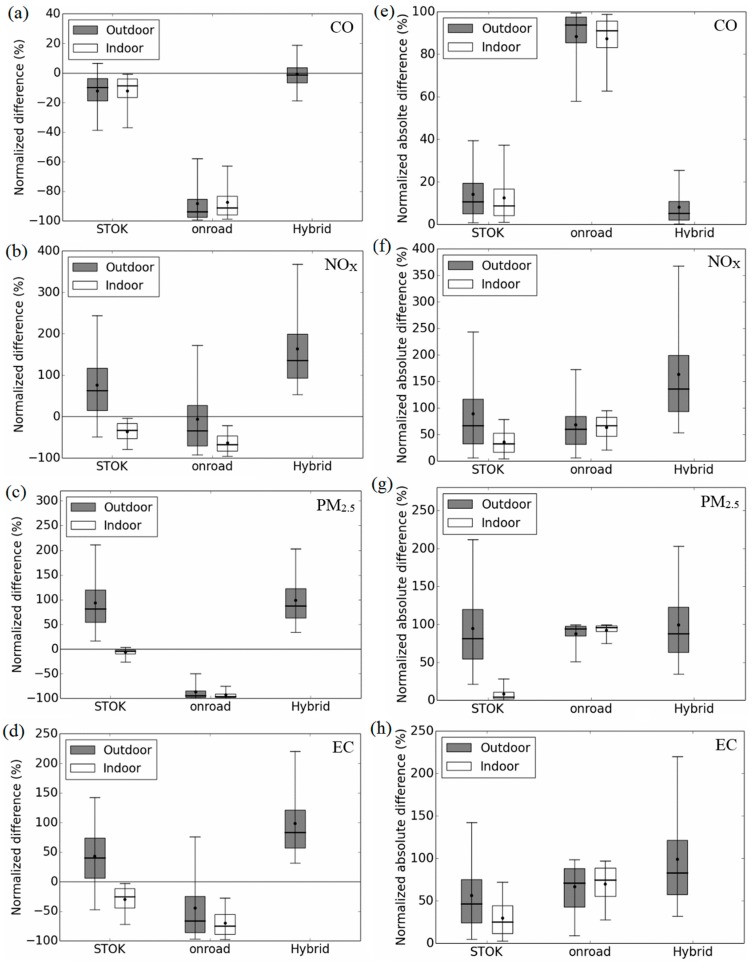
Hourly normalized difference (*ND*, **left panels**) and hourly normalized absolute difference (*NAD*, **right panels**) for each Census block for *CO* (**a**,**e**); *NO_x_* (**b**,**f**); *PM_2.5_* (**c**,**g**); and *EC* (**d**,**h**). Bottom and top of box represents 25th and 75th percentiles, the line in the middle of the box is the median, the ends of the whisker are the 5th and 95th percentiles, and the dot on the whisker is the mean.

Besides the population exposure error, it is also important to quantify the exposure error at an individual level. We quantify this with *ND* (Figure 7 left panel) and *NAD* (Figure 7 right panel). For all pollutants except for *CO*, all outdoor metrics (dark boxes) perform poorly. For example, the average *ND* and *NAD* is 175% with the outdoor hybrid for *NO_x_*. Further, all outdoor metrics have shown wider 90% range (Figure 7 whiskers); so for some Census block, *ND* and *NAD* can be up to 375% for *NO_x_*. From the population exposure error in the previous paragraph, one would expect that the indoor on-road would perform better for *NO_x_* and *EC*. However, for these two pollutants, *ND* and *NAD* indicate that indoor STOK yields lower error (average *ND* ~−25% and *NAD* ~25%) compared to indoor on-road (average *ND* ~−75% and *NAD* ~75%). The disagreement between population and individual exposure error is because although the indoor on-road can capture the locations of the hotspot, the concentration is still too low to represent the true exposure. For *CO*, the best performance is with outdoor hybrid metric (average *ND* ~0% and *NAD* ~10%). Because the penetration factor for *CO* is 1 and the indoor deposition rate is 0, the indoor and outdoor concentration differ less from each other, although *NAD* can still be up to 30% (Figure 7). For *PM_2.5_*, agreeing with the population exposure, the indoor STOK concentration gives the lowest error (average *ND* ~0% and *NAD* ~5%). This is because of the relatively lower contribution from the on-road source for *PM*_2.5_. However, it is worth noting that the error can sometimes be large (up to 25%), indicating on-road source still plays an important role for the near-road population exposure.

## 4. Discussion and Limitations

To prevent bias due to spatial variation [50], many health studies characterized exposure using space-time kriging [13,14,15]. Space-time kriging technique, when lacking adequate number of monitors, may fail to capture a concentration hotspot in microenvironments such as locations found near roadways. Further, using ambient concentrations to represent exposure can introduce exposure error because people spend more time indoors [8,9,10]. These findings motivated this study to quantify the associated potential exposure error to reduce the possible bias in future epidemiological analysis for the CADEE study.

From our analysis, the suitability of an exposure metric to represent a pollutant depends on the pollutant’s three major characteristics: (1) Penetration factor; (2) Deposition rate; and (3) Key source contributor. CO, selected as a “control group” because of its high penetration factor and low deposition rate, is less affected by the indoor infiltration mechanism and thus AER. AER affects all other pollutants’ block-to-block variability although may not be significant when the input outdoor metric’s spatial variability is large. Nevertheless, this small change in spatial variation (~10%) can cause error in population exposure. This is evident in the first “experiment group” where for pollutants with slightly lower penetration factor and higher deposition rate (such as NO*_x_* and EC), the outdoor hybrid, as an input for computing indoor hybrid, produces 20% and 15% more error (Table 4 and Table 6) than indoor on-road. For the second “experiment group” where pollutant is dominated by background (*i.e.*, PM_2.5_), the indoor STOK (Table 5) yields less error for population exposure. It is worth noting that all outdoor metrics cause high exposure error at the population level. This highlights the importance of AER for pollutants with lower penetration factor and higher deposition rate.

At an individual level, CO and PM_2.5_ agree with the results in population exposure error and can be best described by outdoor hybrid and indoor STOK, respectively. For CO, because the infiltration causes little effect on concentration, the concentration has to be characterized by both background and on-road component. For PM_2.5_, although indoor STOK causes little error, that error can still be up to 25%, indicating the importance of on-road emission. This was not seen in another study where concentration was modeled at zip code [7], indicating the necessity to model the concentration at fine spatial resolution. For NO*_x_* and EC, although STOK-based indoor metric gives relatively less error than other metrics, the error is still high (up to 75%) because on-road emission contributes a large portion (38% and 46%) to the total concentration. In terms of individual exposure error, both background and on-road component should be considered.

There are several limitations in this work. First, this study does not consider window opening since data were unavailable. A previous study evaluated the LBL model and another model (LBLX), which extends the LBL model to include natural ventilation from window opening. Based on AER measurements from homes in central North Carolina across four seasons, the LBL and LBLX models had similar uncertainties for days with open windows. Therefore, we do not expect a substantial effect from not including window opening in our study [41]. Secondly, this work does not consider indoor pollutant sources, which could lead to under-prediction for total exposure. Thirdly, since the local source considered in this study focused only on on-road emission, future research should also include other sources such as power plants and other industrial sources in the study area.

## 5. Conclusions

We have provided a comprehensive comparison of multiple tiered exposure metrics and quantified potential exposure error at both population and individual level at 16,095 Census blocks of three Counties in North Carolina for CO, NO*_x_*, PM_2.5_, and elemental carbon (EC) during 2012. These metrics include ambient background concentration from space-time ordinary kriging (STOK), ambient on-road concentration from the Research LINE source dispersion model (R-LINE), hybrid concentration combining STOK and R-LINE, and their associated indoor concentrations from an indoor infiltration mass balance model. We achieved this comprehensive comparison—the main novelty of this study—by combining the different models to obtain spatiotemporally refined outdoor and indoor concentrations. With the examples for the four pollutants, we identified the key factors that can cause the exposure error. Using hybrid-based indoor concentration as the standard, the comparison showed that outdoor STOK metrics yielded large error at both population (67% to 93%) and individual level (average bias between −10% to 95%). For pollutants with significant contribution from on-road emission (EC and NO*_x_*), the on-road based indoor metric performs the best at the population level (error less than 52%). At the individual level, however, the STOK-based indoor concentration performs the best (average bias below 30%). For PM_2.5_, due to the relatively low contribution from on-road emission (7%), STOK-based indoor metric performs the best at both population (error below 40%) and individual level (error below 25%). Finally, the AER calculation in this study, to our knowledge, is the first one using actual house information instead of on-site survey, and at such a refined spatial resolution. This unique approach, along with the comprehensive results from this study provides an opportunity for future researchers to conduct large-scale health studies by selecting appropriate exposure metrics and reduce potential bias in exposure characterization.

## Supplementary Files

Supplementary File 1
