# Supplementary material for: Comparison of Highly Resolved Model-Based Exposure Metrics for Traffic-Related Air Pollutants to Support Environmental Health Studies
Source: Int J Environ Res Public Health. 2015 Dec 8;12(12):15605–25. doi: 10.3390/ijerph121215007 (PMC4690943; doi:10.3390/ijerph121215007)
Supplement: Supplementary File 1 [file ijerph-12-15007-s001.pdf]

Supplementary Information

Comparison of Highly Resolved Model-Based Exposure Metrics for Traffic-Related Air Pollutants to Support Environmental Health Studies

---

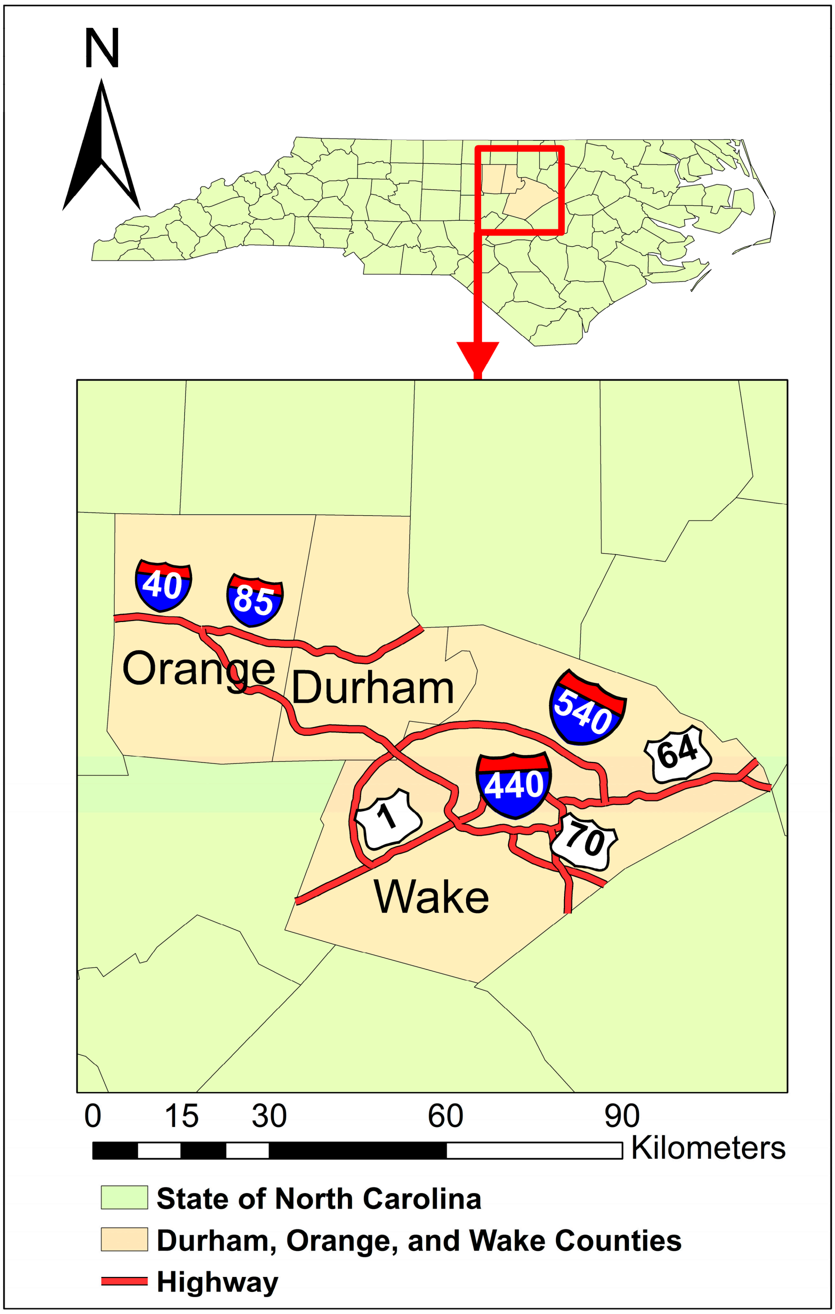

Figure S1. The modeling domain in Durham, Orange, and Wake Counties.

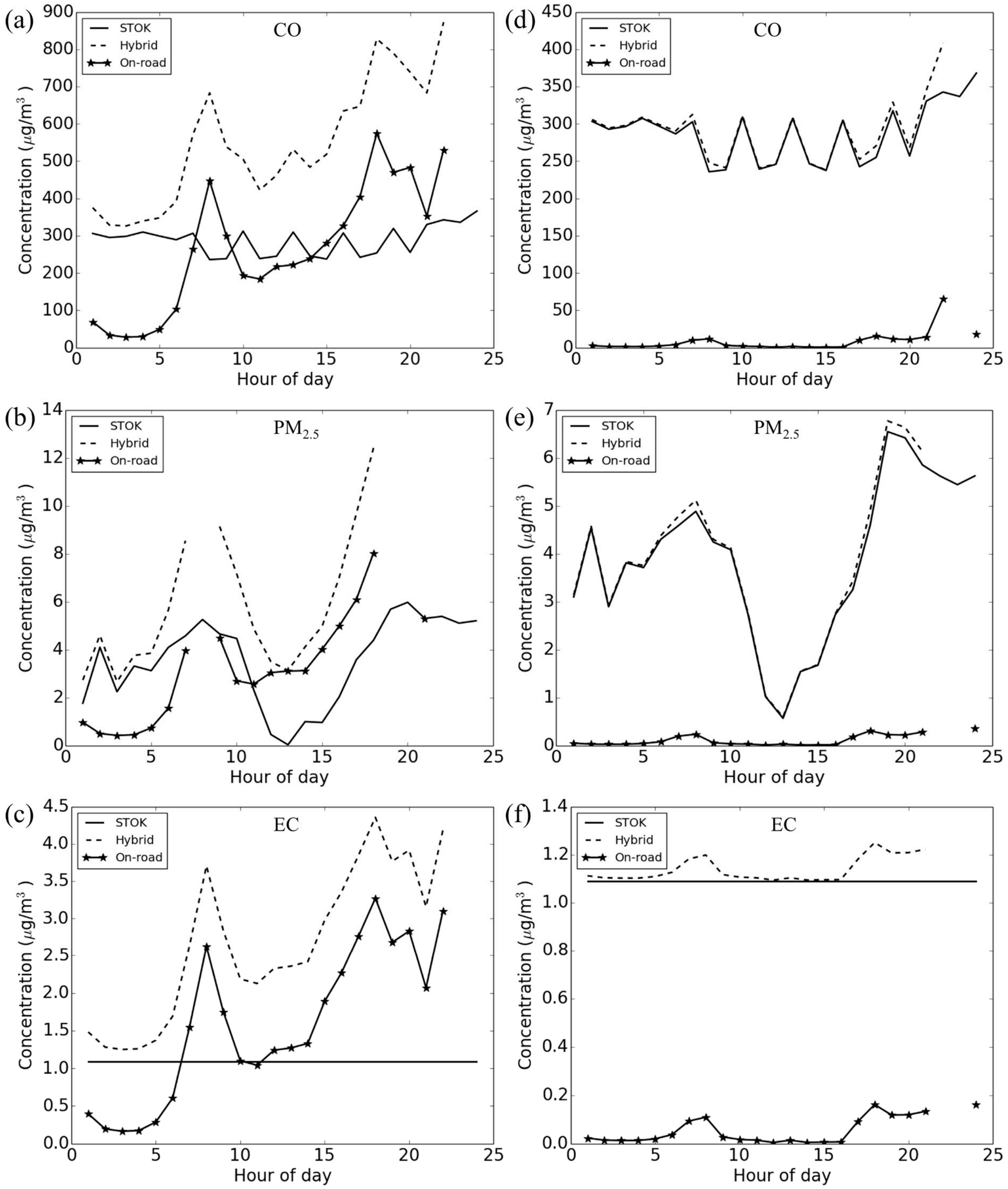

Figure S2. Time series plot on January 3rd for CO (**a**,**d**); $PM_{2.5}$ (**b**,**e**); and EC (**c**,**f**) at a near-road Census block (14.07m from roadway, **left panels**) and a remote Census block (9.57 km from roadway, **right panels**).

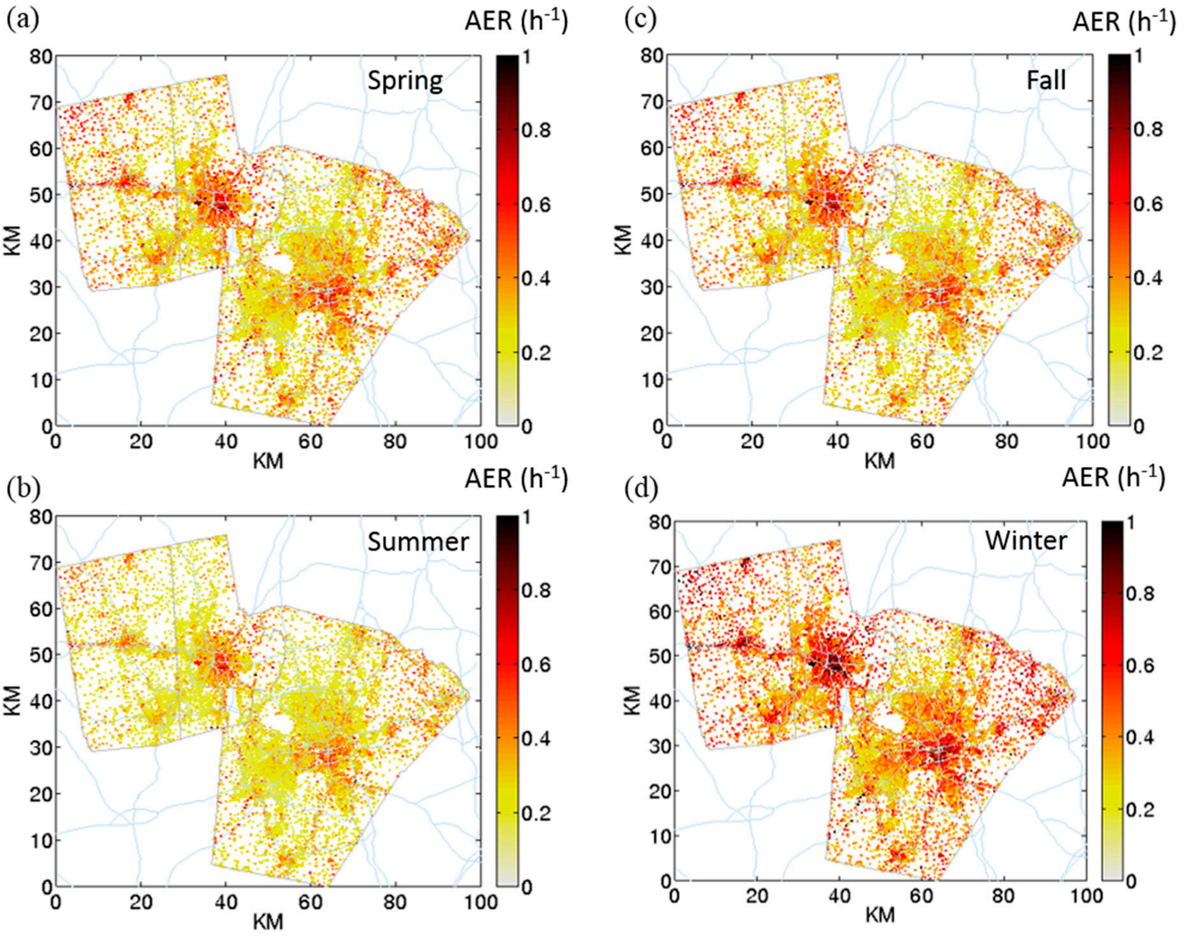

Figure S3. Spatial map for mean AER in (**a**) spring; (**b**) summer; (**c**) fall; and (**d**) winter. The color bar represents unit in (h^{-1}).

Table S1. Stack coefficient k_s in $\frac{\left(\frac{L}{s}\right)^2}{(cm^4 \cdot K)}$.

	House Height (Stories)		
	**One**	**Two**	**Three**
Stack coefficient	0.000145	0.000290	0.000435

Table S2. Wind coefficient k_w in $\frac{\left(\frac{L}{s}\right)^2}{\left(cm^4 \cdot \left(\frac{m}{s}\right)^2\right)}$.

**Shelter Class**	**Number of Stories**		
	**One**	**Two**	**Three**
1	0.000319	0.000420	0.000494
2	0.000246	0.000325	0.000382
3	0.000174	0.000231	0.000271
4	0.000104	0.000137	0.000161
5	0.000032	0.000042	0.000049

Table S3. Local sheltering for LBL model.

Shelter Class [1]	Description [1]	Census Block House Density (House/1000 m^2)
1	No obstructions or local shielding	<0.025
2	Typical shelter for an isolated rural house	0.025–0.03
3	Typical shelter caused by other buildings across street from building under study	0.03–0.5
4	Typical shelter for urban buildings on larger lots where sheltering obstacles are more than one building height away	0.5–1
5	Typical shelter produced by buildings or other structures immediately adjacent(closer than one building height): e.g., neighboring houses on same side of street, trees, bushes, *etc.*	>1

[1] ASHRAE Handbook-Fundamentals, 2009.

© 2015 by the authors; licensee MDPI, Basel, Switzerland. This article is an open access article distributed under the terms and conditions of the Creative Commons Attribution license (http://creativecommons.org/licenses/by/4.0/).
